# A commentary on ‘An anti-infection and biodegradable TFRD-loaded porous scaffold promotes bone regeneration in segmental bone defects - experimental studies’

**DOI:** 10.1097/JS9.0000000000001698

**Published:** 2024-05-22

**Authors:** Yanan Li, Xiaoang Ye, Lianguo Wu

**Affiliations:** a The Second Clinical Medical College of Zhejiang Chinese Medical University; b Department of Orthopaedics, The Second Affiliated Hospital of Zhejiang Chinese Medical University, Hangzhou, Zhejiang, People’s Republic of China

*Dear Editor,*

The recent research paper ‘An anti-infection and biodegradable TFRD-loaded porous scaffold promotes bone regeneration in segmental bone defects - experimental studies’ published in the *International Journal of Surgery* caught our attention^1^. Segmental bone defects are a challenge for clinicians and patients, as they involve long defect distances, slow bone formation, and a long repair period. Long-term observational studies are scarce, and typical clinical methods for treating bone defects include multiple methods, but their disadvantage is the long postoperative treatment process. Many previous studies have only evaluated the bone repair effect of various biomaterials on local bone defects, with limited clinical reference value and a lack of convenient and effective treatment methods. This study is an experimental study that explores the potential application of HA/CMCS/PDA/TFRD scaffolds in the field of segmental bone defects and proposes new treatment options for the treatment of segmental bone defects. Lin *et al*. found that compared to traditional drug therapy for segmental bone defects, HA/CMCS/PDA/TFRD porous scaffolds not only exhibit good degradation and drug release performance, reduce gastrointestinal malnutrition, but also promote osteogenic differentiation of bone marrow mesenchymal stem cells, stimulate cell migration and angiogenesis of human umbilical vein endothelial cells (HUVECs), demonstrate good biocompatibility, and have a good effect on osteogenesis and angiogenesis of bone defects. The emergence of new materials is of great significance, filling the gap in the treatment of segmental bone defects in the medical field. Although we deeply appreciate the rigorous efforts and valuable contributions of this research, we have also put forward some constructive suggestions for further improvement.

Firstly, no cell culture was conducted on the scaffold. Simulation of real situations may have an impact. Unlike two-dimensional cell culture, three-dimensional cell culture promotes cell differentiation and tissue growth by using microarray structures and adjusting complex environmental parameters^2^. In fact, compared to two-dimensional cell cultures, cells in a three-dimensional environment are often more susceptible to morphological and physiological changes^3^. This is because the influence of structural interaction and scaffold can guide the behavior of cells. Researchers have found that the geometric structure and composition of this cell scaffold can not only affect gene expression but also enhance intercellular connections^4^. For example, some genes that promote cell proliferation are inhibited in three-dimensional cell culture, thereby avoiding unrestricted proliferation encountered in two-dimensional cell culture^5^. Therefore, there may be differences in experimental results and surgical applications.

Secondly, when evaluating degradation performance, we did not analyze the changes further in the first 2 weeks. Some studies have shown that the pH value initially decreases and then increases within 2 weeks^6^, but the author’s study did not observe this phenomenon, which may be due to a lack of more detailed time division. It is also unknown whether this phenomenon will have an impact on the final result.

In short, segmental bone defects are a challenging problem for clinical doctors, which requires patients to have strong courage and patience to face long-term health problems. The study by Liu *et al*. demonstrated for the first time that HA/CMCS/PDA/TFRD scaffolds have infection resistance and drug-loading ability, which can promote angiogenesis and osteogenesis to promote the healing of segmental bone defects. Significant contributions have been made to the effective treatment of segmental bone defects, benefiting more patients with related diseases. Our suggestion is only to further improve an already excellent research, and we eagerly look forward to the author’s more innovative works in the future.

## Ethical approval

Not applicable.

## Consent

Not applicable.

## Source of funding

Not applicable.

## Author contribution

Y.L., X.Y., and L.W.: put forward the conception and design of the study; Y.L. and X.Y.: collected and analyzed the data; Y.L. and X.Y.: wrote the paper. All the authors drafted and revised the paper. In addition, all authors read and approved the final manuscript.

## Conflicts of interest disclosure

Not applicable.

## Research registration unique identifying number (UIN)

Not applicable.

## Guarantor

Lianguo Wu accepts full responsibility for the work and/or the conduct of the study.

## Data availability statement

This manuscript is a comment. Don’t need a data availability statement. However, all the data from the current study are publicly available.

## Provenance and peer review

Not applicable.
